# Supplementary material for: Study to Investigate the Knowledge of Rare Diseases among Dentists, Orthodontists, Periodontists, Oral Surgeons and Craniomaxillofacial Surgeons
Source: Int J Environ Res Public Health. 2020 Dec 28;18(1):139. doi: 10.3390/ijerph18010139 (PMC7796213; doi:10.3390/ijerph18010139)
Supplement: Supplementary file 1 [file ijerph-18-00139-s001.zip › Supplement_Questionnaire_Categories.pdf]

# Studie zur Untersuchung der Kenntnis zu Seltenen Erkrankungen unter Zahnärzten, Fachzahnärzten und MKG-Chirurgen

## Fragebogen

1. **Allgemeine Informationen**
2. **Kenntnisse zu seltenen Erkrankungen**
3. **Konfrontation/Auseinandersetzung mit seltenen Erkrankungen**
4. **Ausbildung und Fort-/Weiterbildung**
5. **Informationen zu seltenen Erkrankungen**

1. **Allgemeine Informationen**

**Geschlecht**

- o weiblich
- o männlich

**Alter**

- o < 30 Jahre
- o 30-40 Jahre
- o 41-50 Jahre
- o 51-60 Jahre
- o > 60 Jahre

**Wie lange sind Sie im Beruf?**

- o < 5 Jahre
- o 5-10 Jahre
- o 11-15 Jahre
- o 16-20 Jahre
- o > 20 Jahre

**Sie sind?**

- o Zahnarzt/-ärztin ohne Zusatzbezeichnung/-qualifikation
- o Zahnarzt/-ärztin mit Zusatzbezeichnung/-qualifikation
  - o Oralchirurgie
  - o Mund-Kiefer-Gesichtschirurgie
  - o Kieferorthopädie
  - o Andere: __________________________

**An welcher Universität haben Sie studiert?**

- o TH Aachen
- o Charité - Universitätsmedizin Berlin
- o Universität Bonn
- o TU Dresden
- o Universität Düsseldorf
- o Universität Erlangen-Nürnberg
- o Universität Frankfurt
- o Universität Freiburg
- o Universität Gießen
- o Universität Göttingen
- o Universität Greifswald
- o Universität Halle-Wittenberg
- o Universität Hamburg
- o Universität Hannover
- o Universität Heidelberg
- o Universität Jena
- o Universität Kiel
- o Universität Köln
- o Universität Leipzig
- o Universität Mainz
- o Universität Marburg

- Universität München (LMU)
- Universität Münster
- Universität Regensburg
- Universität Rostock
- Universität des Saarlandes
- Universität Tübingen
- Universität Ulm
- Universität Würzburg
- Universität Witten-Herdecke
- Andere: __________________

**Den Großteil Ihrer Zeit arbeiten Sie?**

- in einem Universitätsklinikum
- in einer Privatklinik
- selbstständig in einer Praxis
- in einer Gemeinschaftspraxis
- in einem Medizinischen Versorgungszentrum
- an einem anderen Ort: ___________________________

## 2. Kenntnisse zu seltenen Erkrankungen

**Wie schätzen Sie Ihre Kenntnisse zu seltenen Erkrankungen ein?**
Versuchen Sie, so spontan und intuitiv wie möglich zu antworten.

>————————————————————————————<

sehr gut ungenügend

**Ihrer Meinung nach ist eine seltene Erkrankung…**

- o Ich weiß nicht genau, was eine seltene Erkrankung ist.
- o eine lebensgefährliche chronische Erkrankung, die häufig genetisch bedingt und schwer heilbar ist.
- o eine Erkrankung, von der nicht mehr als 5 von 10.000 Menschen in der EU betroffen sind.
- o eine Erkrankung, von der nicht mehr als 5 von 250.000 Menschen in der EU betroffen sind.

**Wie viel Prozent der seltenen Erkrankungen manifestieren sich im Mund-Kiefer-Gesichtsbereich?**

- o 5%
- o 7,5%
- o 12%
- o 15%
- o 32%

**Welche Thesen zu seltenen Erkrankungen sind Ihrer Meinung nach korrekt?**
Mehrere Antworten sind möglich.

- o Seltene Erkrankungen sind oft chronische Erkrankungen.
- o Der Großteil der seltenen Erkrankungen ist heilbar.
- o Zur Therapie der seltenen Erkrankungen müssen spezielle Arzneimittel entwickelt werden.
- o Es sind 6.000-8.000 seltene Erkrankungen bekannt.
- o Es sind 8.000-12.000 seltene Erkrankungen bekannt.
- o Der Großteil der seltenen Erkrankungen ist genetisch (mit-)bedingt.
- o Seltene Erkrankungen sind nicht vererbbar.
- o Seltene Erkrankungen manifestieren sich vor allem im frühen Kindesalter.
- o In Deutschland leidet etwa jeder 20. Bundesbürger unter einer seltenen Erkrankung.
- o In der Europäischen Union leiden ca. 30 Millionen Menschen unter einer seltenen Erkrankung.
- o ich enthalte mich
- o Andere: ____________________________________________

**Welche der nachfolgend genannten seltenen Erkrankungen, die sich orofazial manifestieren können, kennen Sie?**

- o Down-Syndrom
- o Ehlers-Danlos-Syndrom
- o Ektodermale Dysplasie
- o Epidermolysis bullosa
- o Fetales Alkoholsyndrom
- o Gorlin-Goltz Syndrom
- o Morbus Behcet
- o Morbus Crohn
- o Osteogenesis imperfecta
- o Pemphigoid
- o Pemphigus vulgaris

- Sklerodermie
- Von-Willebrand-Jürgens Syndrom
- X-chromosomale Hypophosphatämie (Phosphatdiabetes)

**Einschätzung: Nach welcher Zeitspanne wird eine seltene Erkrankung mit orofazialer Manifestation nach erstmaligem Auftreten der Symptome als solche diagnostiziert?**

- innerhalb des 1. Monats
- nach 1-6 Monaten
- nach 6 bis 18 Monaten
- nach 1,5 bis 3 Jahren
- nach mehr als 3 Jahren
- keine Einschätzung

## 3. Konfrontation/Auseinandersetzung mit seltenen Erkrankungen

**Würden Sie sagen, dass Sie sich ausreichend mit seltenen Erkrankungen auskennen?**
Versuchen Sie so spontan und intuitiv wie möglich zu antworten.

>——————————————————————————<

ja　　　　　　　　　　　　　　　　　　　　　　überhaupt nicht

**Haben Sie schon einmal eine/n Patientin/en mit einer seltenen Erkrankung, die sich orofazial manifestiert, behandelt / ist ein/e solche/r Patient/in schon einmal bei Ihnen vorstellig geworden?**

- o ja
- o nein
- o keine Angabe

**Haben Sie schon einmal bei der Behandlung eines/r Patienten/in gedacht, es könnte eine seltene Erkrankung mit orofazialer Beteiligung vorliegen?**

- o ja
- o nein
- o keine Angabe

**Haben Sie schon einmal eine seltene Erkrankung, die sich orofazial manifestiert, diagnostiziert?**

- o ja, ein Mal
- o ja, mehrmals
- o nein, noch nie
- o keine Angabe

## 4. Ausbildung und Fort-/Weiterbildung

**Wurde während Ihrer zahnmedizinischen Ausbildung Zeit darauf verwendet, Wissen über seltene Erkrankungen mit orofazialer Manifestation, ihre Diagnostik und Therapie zu erlangen?**

- o ja, es wurde ausreichend Zeit darauf verwendet
- o ja, aber es wurde zu wenig Zeit darauf verwendet
- o ja
- o nein, daher fehlt es mir jetzt an Wissen bezüglich seltener Erkrankungen mit orofazialer Manifestation
- o nein
- o keine Angabe

**Haben Sie sich bereits bezüglich seltener Erkrankungen mit orofazialer Manifestation fortgebildet?**

- o ja
- o nein, aber ich möchte dies gerne tun
- o nein, es besteht kein Interesse
- o keine Angabe

**Wenn ein/e Patient/in mit bekannter orofazialer Manifestation einer seltenen Erkrankung bei Ihnen vorstellig wird, wissen Sie dann, wo Sie wichtige Informationen zu Diagnostik, Krankheitsverlauf und Therapie erhalten können?**

- o ja
- o nein
- o keine Angabe

**Woher beziehen Sie Ihr Wissen zu seltenen Erkrankungen?**

Mehrere Antworten sind möglich.

- o aus dem Studium
- o aus Fortbildungen
- o aus Fachzeitschriften
- o aus fachspezifischen Online-Portalen
- o von Kollegen
- o keine Angabe

## 5. Informationen zu seltenen Erkrankungen

**Benötigen Sie in Ihrem zahnmedizinischen Alltag Informationen zu seltenen Erkrankungen mit orofazialer Manifestation?**

Mehrere Antworten sind möglich.

- o ja
- o ja, aber ich weiß nicht, wo ich diese Informationen erhalten kann
- o ja, aber ich habe leider keine Zeit für die Recherche
- o nein, ich bin ausreichend informiert
- o nein, da ich nicht an Informationen zu seltenen Erkrankungen mit orofazialer Manifestation interessiert bin
- o keine Angabe

**Welche der unten genannten Organisationen, Websites und Informationsquellen zu seltenen Erkrankungen mit orofazialer Manifestation kennen Sie?**

Mehrere Antworten sind möglich.

- o ROMSE e.V.
- o Orphanet
- o NAMSE (Nationales Aktionsbündnis für Menschen mit Seltenen Erkrankungen)
- o ACHSE e.V. (Allianz Chronischer Seltener Erkrankungen)
- o Keine

**Ich benötige Informationen zu seltenen Erkrankungen mit orofazialer Manifestation bezüglich …**

Mehrere Antworten sind möglich.

- o Inzidenz und Prävalenz
- o Letalität und Mortalität
- o Behandlungsmodalitäten
- o Relevanter Medikationen

**Erachten Sie, als Zahnmediziner, es als wichtig, Kenntnisse zu seltenen Erkrankungen, die sich orofazial manifestieren, zu haben?**

Mehrere Antworten sind möglich.

- o ja, ich erachte es als sehr wichtig
- o ja, diese Kenntnisse sind besonders differentialdiagnostisch von Bedeutung
- o man sollte davon gehört haben
- o nein, es ist absolut unwichtig
- o nein, denn seltene Erkrankungen spielen im zahnmedizinischen Berufsalltag so gut wie gar keine Rolle

## Punktesystem und Einteilung in Kategorien

Für jede richtige Antwort, bekannte RD, Informationsquelle oder benötigte Information und die Antwort „ja“ wird 1 Punkt vergeben, für jede falsche Antwort, „nein“, „keine“, „keine Angabe“ oder Auslassen einer Frage werden 0 Punkte vergeben.

Aus der Gesamtpunktzahl ergeben sich für die jeweiligen Abschnitte folgende mögliche Einteilungen in Kategorien:

**Abschnitt 2+3 „Kenntnisse zu Seltenen Erkrankungen“**

| Kategorie | Punktzahl | Bedeutung |
|---|---|---|
| **1** | **0-10** | **KEINE Kenntnis zu s.E.** |
| **2** | **11-16** | **GERINGE Kenntnis zu s.E.** |
| **3** | **17-22** | **GUTE Kenntnis zu s.E.** |
| **4** | **23-28** | **SEHR GUTE Kenntnis zu s.E.** |

**Abschnitt 4 „Ausbildung und Fort-/Weiterbildung“**

| Kategorie | Punktzahl | Bedeutung |
|---|---|---|
| **1** | **0- 3** | **KEINE/GERINGFÜGIGE Ausbildung / Fort-/Weiterbildung** |
| **2** | **4- 6** | **MÄßIGE Ausbildung / Fort-/Weiterbildung** |
| **3** | **7-10** | **GUTE/SEHR GUTE Ausbildung / Fort-/Weiterbildung** |

**Abschnitt 5 „Informationsbedarf zu Seltenen Erkrankungen“**

| Kategorie | Punktzahl | Bedeutung |
|---|---|---|
| **1** | **0- 5** | **GERINGER Bedarf an Informationen zu s.E.** |
| **2** | **6-10** | **MÄßIGER Bedarf an Informationen zu s.E.** |
| **3** | **11-14** | **GROßER Bedarf an Informationen zu s.E.** |
