# Supplementary material for: Study to Investigate the Knowledge of Rare Diseases among Dentists, Orthodontists, Periodontists, Oral Surgeons and Craniomaxillofacial Surgeons
Source: Int J Environ Res Public Health. 2020 Dec 28;18(1):139. doi: 10.3390/ijerph18010139 (PMC7796213; doi:10.3390/ijerph18010139)
Supplement: Supplementary file 1 [file ijerph-18-00139-s001.zip › Questionnaire_english version.docx]

**Study to investigate the Knowledge about Rare Diseases among Dentists, Specialist Dentists and Craniomaxillofacial Surgeons**

**Questionnaire**

**1. General Information**

**2. Knowledge about rare diseases**

**3. Confrontation/Experience with rare diseases**

**4. Education/Continuing Education**

**5. Information about rare diseases**

1. **General Information**

**Gender**

- male
- female

**Age**

- < 30 years
- 30-40 years
- 41-50 years
- 51-60 years
- > 60 years

**How long have you been practicing your profession?**

- < 5 years
- 5-10 years
- 11-15 years
- 16-20 years
- > 20 years

**You are?**

- Dentist without specialization
- Dentist with specialization
  - Oral surgery
  - Oral- and craniomaxillofacial surgery
  - Orthodontics
  - others: _________________

**Which university did you study at?**

- TH Aachen
- Charité - Universitätsmedizin Berlin
- University of Bonn
- TU Dresden
- University of Düsseldorf
- University of Erlangen-Nürnberg
- University of Frankfurt
- University of Freiburg
- University of Gießen
- University of Göttingen
- University of Greifswald
- University of Halle-Wittenberg
- University of Hamburg
- University of Hannover
- University of Heidelberg
- University of Jena
- University of Kiel
- University of Köln
- University of Leipzig
- University of Mainz
- University of Marburg
- University of München (LMU)
- University of Münster
- University of Regensburg
- University of Rostock
- University of des Saarlandes
- University of Tübingen
- University of Ulm
- University of Würzburg
- University of Witten-Herdecke
- others: _________________

**Most of your time you work at …?**

- a university dental hospital
- a private hospital
- self-employed in a medical practice
- a joint practice
- a medical care center
- others: __________________

1. **Knowledge about rare diseases**

**How do you assess your knowledge about rare diseases?**

Please try to answer as intuitively as possible.

>

<

very good inadequate

**In your opinion a rare disease is…?**

- I do not know exactly what a rare disease is.
- a life-threatening chronic disease, which is often genetically determined and difficult to cure.
- a disease of which no more than 5 of 10,000 people in the EU are affected.
- a disease of which no more than 5 of 250,000 people in the EU are affected.

**How many percent of rare diseases manifest themselves in the craniomaxillofacial region?**

- 5%
- 7.5%
- 12%
- 15%
- 32%

**Which statements on rare diseases do you think are correct?**

You can choose several answers.

- Rare diseases are often chronic diseases.
- Most rare diseases are curable.
- Special drugs must be developed for the treatment of rare diseases.
- There are 6000 to 8000 rare diseases.
- There are 8000 to 12,000 rare diseases.
- Most rare diseases are genetically (co-)related.
- Rare diseases are not hereditary.
- Rare diseases manifest themselves primarily in early childhood.
- In Germany about every 20th German citizen suffers from a rare disease.
- In the European Union, approximately 30 million people suffer from a rare disease.
- I abstain.
- Others: ________________________________

**Which of the following rare diseases, which can manifest themselves orofacially, do you know?**

- Down Syndrome
- Ehlers-Danlos-Syndrome
- Ectodermal-Dysplasia
- Epidermolysis-Bullosa
- Fetal alcohol syndrome
- Gorlin-Goltz-Syndrome
- Behçet’s disease
- Crohn’s disease
- Osteogenesis imperfecta
- Bullous pemphigoid
- Pemphigus vulgaris
- Scleroderma
- Von Willebrand-Jürgens-Syndrome
- X-linked-Hypophosphataemia

**Assessment: How long does it take for a rare disease with orofacial manifestation to be diagnosed as such after the first appearance of symptoms?**

- It is diagnosed within the first month.
- … after 1 to 6 months.
- … after 6 to 18 months.
- … after 1.5 to 3 years.
- … after more than 3 years.
- no estimation

1. **Confrontation/Experience with rare diseases**

**Do you think your knowledge about rare diseases is sufficient?**

Please try to answer as intuitively as possible.

>

**<**

yes not at all

**Have you ever treated a patient affected by a rare disease/have you ever seen such a patient before?**

- yes
- no
- no information

**Have you ever thought a rare disease with orofacial manifestation may be present while treating a patient?**

- yes
- no
- no information

**Have you ever diagnosed a rare disease which manifests itself orofacially?**

- yes, once
- yes, several times
- no, never
- no information

**4. Education/Continuing Education**

**During your dental education, has there any time been spent on acquiring knowledge about rare diseases with orofacial manifestations, their diagnostics and therapy?**

- Yes, sufficient time was spent on acquiring information about them.
- Yes, but too little time was spent on acquiring information about them.
- yes
- No. That is why I have a lack of knowledge about rare diseases with orofacial manifestations.
- no
- no information

**Have you already attended training courses with focus on rare diseases with orofacial manifestations?**

- yes
- No, but I would like to.
- No, I am not interested in training courses with focus on rare diseases.
- no information

**Do you know where to get information about diagnostics, course of disease and therapy when treating a patient affected by a rare disease with orofacial manifestations?**

- yes
- no
- no information

**Which of the following possible sources do you use as source of knowledge about rare diseases?**

You can choose several answers.

- the studies
- further education
- trade journals
- subject-specific online portals
- colleagues
- no information

**5. Information about rare diseases**

**Do you need information concerning rare diseases with orofacial manifestations in your everyday dental practice?**

You can choose several answers.

- yes
- Yes, but I do not know where to get this information.
- Yes, but unfortunately, I do not have time for research.
- No, I am sufficiently informed.
- No, because I am not interested in information concerning rare diseases with orofacial manifestations.
- no information

**Which of the following organizations, websites and sources of information on rare diseases with orofacial manifestations do you know?**

You can choose several answers.

- ROMSE e.V.
- Orphanet
- NAMSE (Nationales Aktionsbündnis für Menschen mit Seltenen Erkrankungen)
- ACHSE e.V. (Allianz Chronischer Seltener Erkrankungen)
- none

**I need information about rare diseases with orofacial manifestations concerning …**

You can choose several answers.

- incidence and prevalence
- lethality and mortality
- treatment modalities
- relevant medications

**Do you, as a dentist, consider it important to have knowledge about rare diseases that manifest orofacially?**

You can choose several answers.

- Yes, I consider it to be very important.
- Yes, knowledge about rare diseases has an important differential diagnostic significance.
- One should have heard about rare diseases.
- No, it is unimportant.
- No, rare diseases virtually play no role at all in everyday dental practice.

**Scoring system and division into categories**

For each correct answer, known rare disease, source of information or required information and answer “yes”, 1 point is assigned; for each wrong answer, “no”, “none”, “no information” or omitted question, 0 points are awarded.

The evaluation of the answers can thus be based on the total number of points achieved for the respective section and can be classified according to a division into the following categories:

**Sections 2+3 “Knowledge about rare diseases“**

| Category | number of points | meaning |
| --- | --- | --- |
| **1** | **0-10** | **no knowledge** |
| **2** | **11-16** | **little knowledge** |
| **3** | **17-22** | **good knowledge** |
| **4** | **23-28** | **very good knowledge** |

**Section 4 “Education/Continuing education“**

| Category | number of points | meaning |
| --- | --- | --- |
| **1** | **0- 3** | **no/little education** |
| **2** | **4- 6** | **moderate education** |
| **3** | **7-10** | **(very) good education** |

**Section 5 “Information needs on rare diseases“**

| Category | number of points | meaning |
| --- | --- | --- |
| **1** | **0- 5** | **no information needs** |
| **2** | **6-10** | **little information needs** |
| **3** | **11-14** | **considerable information needs** |
